# Surface‐based morphometry of the corpus callosum in young children of ages 1–5

**DOI:** 10.1002/hbm.26693

**Published:** 2024-06-25

**Authors:** Niharika Gajawelli, Athelia Paulli, Sean Deoni, Natacha Paquette, Danielle Darakjian, Carlos Salazar, Douglas Dean, Jonathan O'Muircheartaigh, Marvin D. Nelson, Yalin Wang, Natasha Lepore

**Affiliations:** ^1^ CIBORG Lab, Department of Radiology Children's Hospital Los Angeles Los Angeles California USA; ^2^ Department of Pediatrics Warren Alpert Medical School at Brown University Providence Rhode Island USA; ^3^ Bill & Melinda Gates Foundation Seattle Washington USA; ^4^ Department of Psychology CHU Sainte‐Justine Montreal Quebec Canada; ^5^ College of Medicine California Northstate University Elk Grove California USA; ^6^ Department of Biomedical Engineering University of Southern California Los Angeles California USA; ^7^ Waisman Laboratory for Brain Imaging and Behavior University of Wisconsin Madison Madison Wisconsin USA; ^8^ Department of Neuroimaging King's College London London UK; ^9^ Department of Pediatrics University of Southern California Los Angeles California USA; ^10^ Department of Radiology Children's Hospital Los Angeles Los Angeles California USA; ^11^ Department of Computer Science Arizona State University Tempe Arizona USA

## Abstract

The corpus callosum (CC) is a large white matter fiber bundle in the brain and is involved in various cognitive, sensory, and motor processes. While implicated in various developmental and psychiatric disorders, much is yet to be uncovered about the normal development of this structure, especially in young children. Additionally, while sexual dimorphism has been reported in prior literature, observations have not necessarily been consistent. In this study, we use morphometric measures including surface tensor‐based morphometry (TBM) to investigate local changes in the shape of the CC in children between the ages of 12 and 60 months, in intervals of 12 months. We also analyze sex differences in each of these age groups. We observed larger significant clusters in the earlier ages between 12 v 24 m and between 48 v 60 m and localized differences in the anterior region of the body of the CC. Sex differences were most pronounced in the 12 m group. This study adds to the growing literature of work aiming to understand the developing brain and emphasizes the utility of surface TBM as a useful tool for analyzing regional differences in neuroanatomical morphometry.

## Graphical abstract

We use morphometric measures including surface tensor‐based morphometry to investigate local changes in shape and sex differences of the corpus callosum (CC) in children between the ages of 12 and 60 months. We observed significant clusters in 12 v 24 m and between 48 v 60 m and localized differences in the anterior region of the body of the CC.

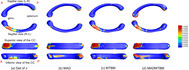

## 1 INTRODUCTION

An increasing number of studies report that cognitive and behavioral deficits in neurodevelopmental and psychiatric disorders correlate with subtle structural changes in the corpus callosum (CC), including autism (Gilmore et al., 2007; Pujol et al., 1993), schizophrenia (Chura et al., 2010; Giedd et al., 1996; Matsuzawa, 2001), and attention‐deficit disorder (Giedd et al., 1994). Other studies have also shown sex differences in the subregions of the CC and have associated these differences with morphometric abnormalities in neuropsychiatric disorders, including autism spectrum disorders, dyslexia, attention‐deficit disorder, and Tourette syndrome (Paul, 2010; Von Plessen et al., 2002). These findings underscore our need to better understand the structural development of the corpus callosum and how it potentially contributes to aspects of neurodevelopmental or psychiatric disorders.

The corpus callosum is the largest white matter fiber bundle in the brain and integrates motor, sensory, and cognitive processes between the two hemispheres of the brain. The CC is generally divided into regions including the genu (the most anterior part of the CC), the rostrum (the inferior part of the CC after the anterior bend), the body, the isthmus, and the splenium (the posterior part of the CC) (Knyazeva, 2013). While there are no macroscopic anatomical landmarks delineating exact callosal subregions, there are two widely used subregional parcellation schemes, both of which rely on geometric parcellation of the midsagittal corpus callosum according to functional specialization: the Witelson parcellation scheme and the Hofer and Frahm parcellation scheme (Hofer & Frahm, 2006; Cover et al., 2017). The Witelson parcellation scheme, first proposed in 1989 and based primarily on postmortem histological data collected from non‐human primates, subdivides the CC into five vertical segments based on fractional divisions along the maximum anterior–posterior length of the structure (Witelson, 1989). Notably, the Witelson parcellation scheme does not accurately reflect CC regional differences at a cellular level, based on examinations of callosal fiber composition via light microscopic examination (Aboitiz et al., 1992; Hofer & Frahm, 2006).

Hofer and Frahm proposed an updated midsagittal CC parcellation scheme, basing their five suggested vertical subdivisions on differences in cortical connectivity of the CC, ascertained using diffusion tensor imaging based fiber tractography in healthy human subjects (Hofer & Frahm, 2006). Similar to the Witelson parcellation, the Hofer and Frahm parcellation scheme establishes a geometric baseline along the maximum anterior–posterior length of the CC, upon which five vertical subdivisions are delineated. The Hofer and Frahm parcellation defines region I (genu) as the anterior‐most 1/6th of the CC, with thinner axonal fibers projecting into prefrontal regions. The remaining anterior half of the CC comprises region II, which contains fibers projecting to premotor and supplementary motor cortical areas. The posterior half minus the posterior‐most 1/3rd makes up region III, which contains fibers projecting to the primary motor cortex. Axonal fibers in regions II and III are comparatively thicker relative to axons in regions I, IV, and V (Aboitiz et al., 1992; Hofer & Frahm, 2006). Region IV consists of the posterior‐most 1/3rd minus the posterior‐most 1/4th and contains primary sensory fibers. The posterior‐most 1/4th comprises region V (splenium), through which parietal, temporal, and occipital fibers cross the CC (Hofer & Frahm, 2006). This paper honours the Hofer and Frahm parcellation scheme when discussing subregional CC cortical connectivity.

While the CC contains heterotopic connections that can help integrate information between nonidentical regions of the brain, the majority of the axonal projections of the corpus callosum are homotopic, connecting homologous areas between the two hemispheric regions (Giedd, Blumenthal, Jeffries, Castellanos, et al., 1999; Giedd, Blumenthal, Jeffries, Rajapakse, et al., 1999; Hofer & Frahm, 2006). The anterior callosal fibers connecting the frontal lobes transfer motor information between the two hemispheres, helping to coordinate motor functions. The anterior CC also contains thinner associative fibers that facilitate coordination and communication between nonhomologous regions of the cerebral cortex, which is essential for complex cognitive processing (Fabri & Polonara, 2023). The posterior callosum (splenium) fibers connect posterior regions of the cerebral cortex, including the posterior parietal, temporal and occipital lobes, and play a role in the transfer of multimodal sensory information between the two hemispheres. Specifically, auditory information integration is associated with the isthmus of the CC and visual information integration is associated with the splenium (Tanaka‐Arakawa et al., 2015; Xu et al., 2013).

The CC is commonly investigated using diffusion weighted MRI scans, a technique useful to study white matter (WM) in the brain. Through measures of fractional anisotropy, diffusion tensor imaging can show the extent of diffusion in WM tracts such as the CC (de Lacoste et al., 1985; Hofer & Frahm, 2006). Despite accessibility of modalities to investigate this structure, much has yet to be uncovered about the normal structural pediatric development of the CC. This is especially true in children under age 5, due to the limited number of large‐scale systematic neuroimaging studies in early childhood.

On T1‐weighted MRI scans, the CC can be seen clearly on a midsagittal MRI slice due to the WM contrast produced by the myelin sheath that wraps around the axonal fibers. During early brain development, the CC myelinates at different rates postnatally, as examined in a study of autopsied infants (Gilles & Nelson, 2012). For instance, the rostrum myelinates later than other callosal regions, with grossly visible myelin of 50% of cases at 12 months age and myelination continuing after the 2nd year. On the other hand, the body of the CC, with fibers connecting the midportions of the posterior frontal and parietal lobes, showed microscopic myelination at term in 25% of cases. Furthermore, myelination occurred rapidly after 5 months of age, and the body of the CC was grossly myelinated by 13 months of age in 90% of the cases. The same study also showed the splenium, containing fibers connecting the temporal and occipital lobes, had grossly visible myelination in 90% of the cases by 21 months (Gilles & Nelson, 2012). There are a few studies using myelin water fraction (MWF) that show an increasing degree of myelination in children from 3 months to 60 months (Dean et al., 2014; Deoni et al., 2012), as well as a very recent study using longitudinal multimodal imaging to investigate gender sex differences in infants (Schmied et al., 2020).

Other studies on CC development have shown callosal size or thickness changes through analysis of the midsagittal slice. For example, Giedd et al. showed that total midsagittal CC area increased robustly from ages 5–18 years in healthy children and that posterior and mid‐regions of the CC showed larger age‐related changes than anterior regions (Giedd, Blumenthal, Jeffries, Castellanos, et al., 1999; Giedd, Blumenthal, Jeffries, Rajapakse, et al., 1999). In another study that included a younger cohort of subjects, from 1 month of age to adults, it was shown that the developmental trajectory of CC area followed a similar trajectory as the cortex in the first few years of life regardless of sex (Tanaka‐Arakawa et al., 2015). The authors also showed that the females had higher ratios of total CC, genu, posterior midbody, and splenium to the brain, compared to males (Tanaka‐Arakawa et al., 2015). In another study exploring sex differences in healthy children aged 8–15 years, callosal thickness increased across the whole surface, except for the rostrum in females (Luders et al., 2010).

The existing literature on anatomical MRI studies (Giedd, Blumenthal, Jeffries, Rajapakse, et al., 1999; Giedd et al., 1996; Tanaka‐Arakawa et al., 2015) that specifically examined the healthy development of the CC used the midsagittal slice size for analysis. While such analysis offers important information regarding development, the CC is a volumetric structure that extends out laterally from the midsagittal slices. Hence, three‐dimensional surface‐based measures, combined with more traditional measures such as thickness, will add value in mapping out CC development more comprehensively. For example, surface‐based morphometry analysis has shown differences in the morphological development of the splenium of the CC (Shi et al., 2015; Xu et al., 2013) in a study investigating CC differences in the congenitally blind, late blind (onset >8 years), and sighted subjects (Shi et al., 2015; Xu et al., 2013).

In this study, we use several surface‐derived measures including multivariate surface tensor‐based morphometry (mTBM) and medial axial distance (MAD) to investigate group differences in the CC (Lepore et al., 2008; Wang et al., 2010). MTBM utilizes the Jacobian matrix or measures from the Jacobian matrix, computed at each vertex of the displacement field between a subject image and template image, for group comparisons. MAD is the distance between the mid‐line of the CC and each vertex on the surface of the structure. These methods have been used in previous studies and show superior detection of regional differences in the morphology of brain surfaces and volumes between subject groups (Wang, Panigrahy, et al., 2011; Wang, Song, et al., 2011). Additionally, combined measures of mTBM along MAD reveal significantly improved statistical power (Shi et al., 2014, 2013; Wang, Song, et al., 2011; Wang et al., 2013). While this method has proved to be sensitive to differences between various populations, it has never been used to investigate localized changes that occur in early brain development of the corpus callosum. The age range under 60 months is especially important since the brain develops to 90% of its final volume by age 5 (Brown & Jernigan, 2012; Courchesne et al., 2000; Iwasaki et al., 1997; Lenroot & Giedd, 2006). As the CC is a central structure of the brain, we expect to see significant development.

In this paper, we investigate the CC development in children aged 12 to 60 months in 12 month increments using combined thickness and surface‐based morphometry measures to extract localized differences that might be associated with early brain development. Additionally, we also analyze sex differences in each age group. There is evidence of sexual dimorphism in literature; however, studies have also shown no sex related differences. The lack of consistency in previous studies may be due to brain size, the age range investigated, the variability in the subjects used, as well as due to differences within a particular brain structure. Our goal here is to add to the existing body of work in understanding sex differences by providing surface‐based morphometry results in this unique age range of 12–60 months.

## 2 METHODS AND MATERIALS

### 2.1 Data

Our dataset comprised brain volumes from 160 healthy subjects ranging from 12 to 60 months (mean and standard deviation in weeks shown in Table 1 below) of age from the Advanced Baby Imaging Lab database (www.babyimaginglab.com) collected longitudinally. All available data were quality checked for artifacts, such as excessive motion that would hinder the delineation of the CC. In this process, the following number of subjects were excluded: 4 in the 12 m cohort, 3 in the 24 month cohort, 3 in the 36 month cohort, 3 in the 48 month cohort, and 7 in the 60 month cohort. Those passing quality check during the original analysis was used, and the total number of subjects used in the study are displayed in Table 1. Neuroimaging data were collected for research purposes and not for medical reasons. Inclusion criteria for the participants were: singleton birth between 37 and 42 weeks gestation with no abnormalities on fetal ultrasound and no reported history of neurological events or disorders in the infant. Remer et al. list further details of the population scanned and protocols applied (Remer et al., 2017). Children included in the dataset were further divided into 5 age groups (12 months, 24 months, 36 months, 48 month, and 60 months). Sex differences were investigated for each of these groups. The distribution of subjects is shown in Table 1.

**TABLE 1:** Distribution of the subjects in different age groups.

Age	Mean age/std. dev (weeks)	Number of subjects	M	F
12 months	53.4/5.3	34	17	16
24 months	102.8/6.7	31	15	16
36 months	153/14.1	31	15	16
48 months	201.8/15.8	29	15	14
60 months	260.7/13.8	33	17	16

Inversion spoiled gradient echo (IR‐SPGR) images were acquired (5° flip angle, TR: 16 ms, TE:6.9 ms, TI:950 ms, voxel resolution 1.2 mm^3^ × 1.2 mm^3^ × 1.2 mm^3^) (Remer et al., 2017). The voxel resolution was maintained across ages by varying the acquisition matrix and field of view according to head size (Deoni et al., 2012; Deoni et al., 2015). Prior to the MRI acquisition, each subject or their guardian was informed of the goals of the study and signed a formal consent. The study was approved by the Institutional Review Board of Brown University. All data were deidentified before preprocessing. Note that the handedness of the subjects was not assessed in this study due to scarcity of handedness information at early ages.

### 2.2 Processing pipeline

**FIGURE 1:**
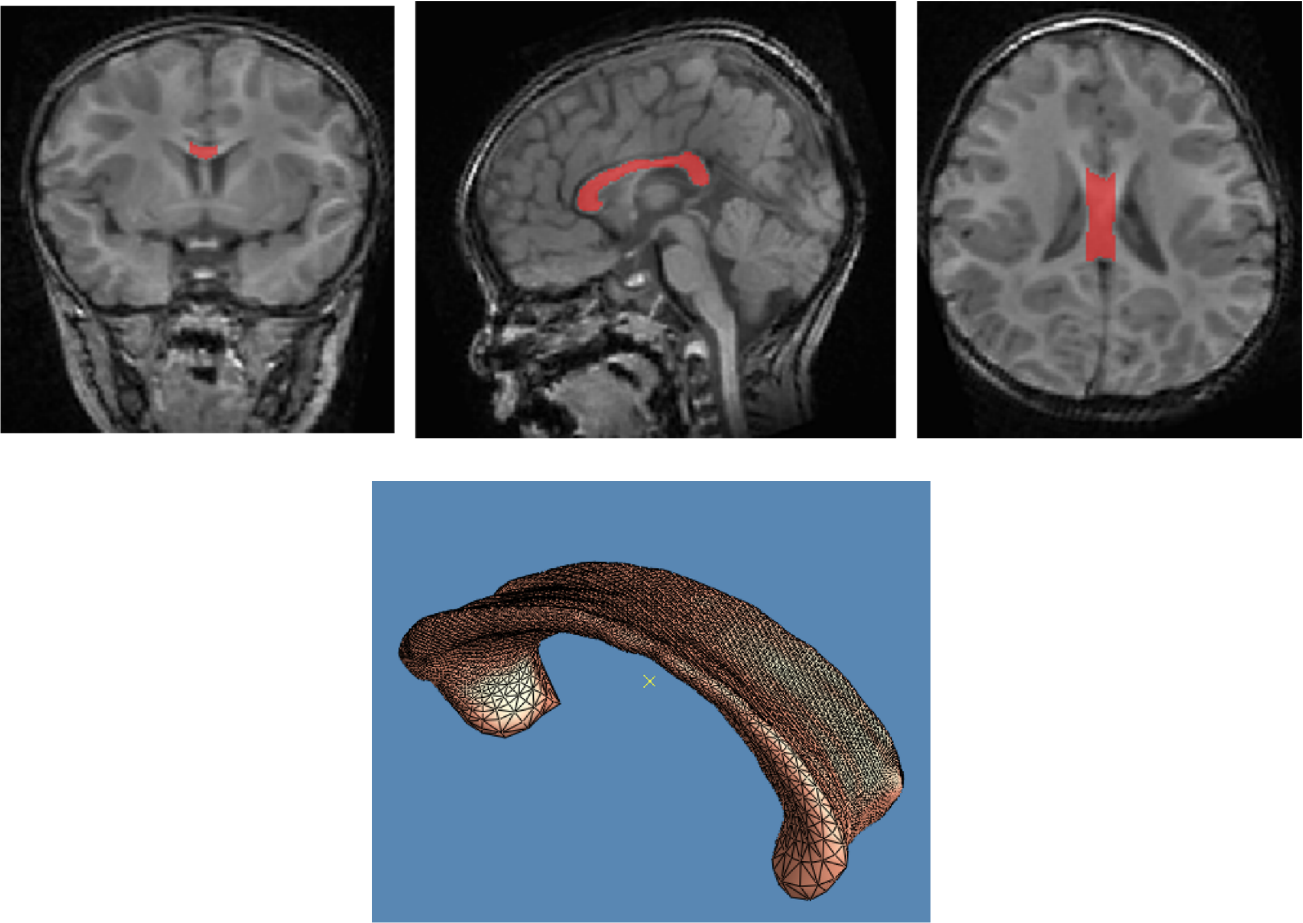
(Top) Coronal, mid‐sagittal, and axial slices displaying the segmented CC. (Bottom) Representation of a tetrahedral mesh generated on the segmentation of a CC.

The T1‐weighted MP‐RAGE scans were first skull stripped using FSL BET (Jenkinson et al., 2005) and then rigidly registered using 6 degrees of freedom to an age‐matched template in the MNI space. The CC was then manually segmented using the ITK‐snap toolkit (http://www.itksnap.org) (Yushkevich et al., 2006). Neuroanatomical references of the corpus callosum were used to determine the segmentation. The CC was segmented on sagittal slices of the brain surrounding the center slice (+/− 6 slices), as shown in the colored region of Figure 1 (top), similar to previous studies (Shi et al., 2015). The CC typically fans out at the genu and splenium, and while the center sagittal slices show the CC very clearly, it was not possible to consistently visually identify where these regions of the CC end on an pediatric MR images. The segmentation protocol was approved by a pediatric neuroradiologist. Therefore, only the mid‐sagittal slices were analyzed for this study, which were consistent across subjects due to the prior registration done to an age‐matched template. All segmentations were done by a single person. After segmentation, 3‐dimensional tetrahedral meshes representing the CC were created using an in‐house conformal mapping program. This program is based on an adaptively sized tetrahedral mesh molding method (Lederman et al., 2011). An example of a mesh is shown in Figure 1.

### 2.3 Experimental design and statistical analysis

#### 2.3.1 Surface registration and multivariate tensor‐based morphometry (mTBM)

Since the CC is long and thin in shape, subcortical surface parameterization algorithms (Styner et al., 2005) such as spherical harmonics (Nitzken et al., 2013) might lead to large distortions in the registration. Therefore, instead of a sphere, the CCs are modeled as surfaces topologically equivalent to cylinders with two open boundaries using the holomorphic one‐form based method (Wang, Panigrahy, et al., 2011). Briefly, after the surface mesh is constructed, biologically valid and consistent landmark curves at the caudal and rostral endpoints of the callosal surface are automatically detected as the extreme points of the CC and marked. The CC was then sliced open, thereby creating a genus‐0 surface with open boundaries. Constrained harmonic mapping was used to achieve one‐to‐one correspondence between the cylindrical plane and each anatomical surface. More details can be found in (Shi et al., 2015). In mTBM, the registration between each subject and the template yields a displacement field. At each vertex, a Jacobian matrix.

$J=\frac{\mathrm{d\phi u}}{\mathrm{du}}\;\frac{\mathrm{d\phi u}}{\mathrm{du}}\;\frac{\mathrm{d\phi v}}{\mathrm{du}}\;\frac{\mathrm{d\phi v}}{\mathrm{dv}}$, can then be computed from the deformation field and parameters from the Jacobian matrix can be used as metrics for subsequent group comparisons.

The determinant of J (det of J) is an important univariate measure of local area changes and their directions (i.e. shrinkage or expansion), however, this gives us an incomplete picture of the CC morphological changes with age. Multivariate measures, such as mTBM, have been shown to give more statistical power compared to univariate measures (Lepore et al., 2008; Wang Phd et al., n.d.; Wang, Song, et al., 2011). Therefore, we used the deformation tensor, used in mTBM, defined by $S=\sqrt{\mathrm{JTJ}}$ ^1/2^, also known as the Cauchy‐Green tensor. On the each vertex of the tetrahedral mesh, the Jacobian is computed by [w3 – w1, w2 – w1][v3 – v1, v2 – v1]^−1^, where [v1, v2, v3] is a triangle on the subject mesh being mapped on a triangle on a template mesh [w1, w2, w3]. Since $S=\sqrt{\mathrm{JTJ}}$ ^1/2^, gives us 3 elements at each vertex, log(S) can be computed to get a 3 × 1 feature vector to use as a multivariate measure (Leporé et al., 2008).

### 2.4 Thickness computation

Medial axial distance (MAD) is one of the most commonly used morphometry measures on surface data (Pizer et al., 1999; Wang, Song, et al., 2011). Specifically, MAD calculated in 3D structure informs us on the entire surface CC and as opposed to solely the midsagittal plane. The radial distance is calculated as the distance from the medial axis to each vertex (Wang, Song, et al., 2011) and the medial axis is found using the mid‐point of the iso‐parametric curves, which are perpendicular to the computed conformal grid. While mTBM is sensitive to changes such as shear along the surface tangent direction, the thickness calculated allows us to investigate change along the surface normal direction. Combining both of these measures, we construct a 4 × 1 multivariate feature vector (Shi et al., 2015; Wang, Song, et al., 2011), called MADMTBM here.

### 2.5 Group statistics

For group comparisons, the Hotelling's T^2^ test, the multivariate generalization of the Student's t‐test (Hotelling, 1931), was applied on sets of values in the Log‐Euclidean space of the deformation tensors. The Mahalanobis distance M, defined below, was used to measure the mean vector differences between age groups.

(1) $$M=\frac{N_{S}N_{T}}{N_{S}+N_{T}}{\left(S-T\right)}^{\prime }\Sigma^{-1}\left(S-T\right)$$

Here N_S_ and N_T_ are the number of subjects in two different groups, S and T are the mean vectors of each group, and sigma is the combined covariance matrix of these two groups (Leporé et al., 2008). To eliminate the assumption for a normal distribution, we used a permutation test, randomly assigning subjects to different groups, and then compared the true labels to the distributions generated (Lepore et al., 2008; Shi et al., 2015). A second permutation test was run to correct for multiple comparisons (Lepore et al., 2008; Leporé et al., 2008; Shi et al., 2015; Wang et al., 2013). The permutations were repeated 10,000 times in both cases.

Four group statistic measures, (det of J, MAD, mTBM, and the combined measure of the latter two, MADMTBM) were calculated between 12 versus 24 months, 24 versus 36 months, 36 versus 48 months, and 48 versus 60 month group cohorts, as well as 12 versus 60 m cohorts to understand where the largest differences lie in our age span.

In addition, the same group statistics were also computed to investigate sex differences within each age group. Due to the superior sensitivity of the MADMTBM measure, as it includes information from the det of J, MAD, and mTBM, and for clarity, only the MADMTBM results are shown for sex.

Finally, to better understand the direction of change, ratio maps for MAD and det of J were calculated between each subsequent age group, as well as between the sexes.

## 3 RESULTS

**FIGURE 2:**
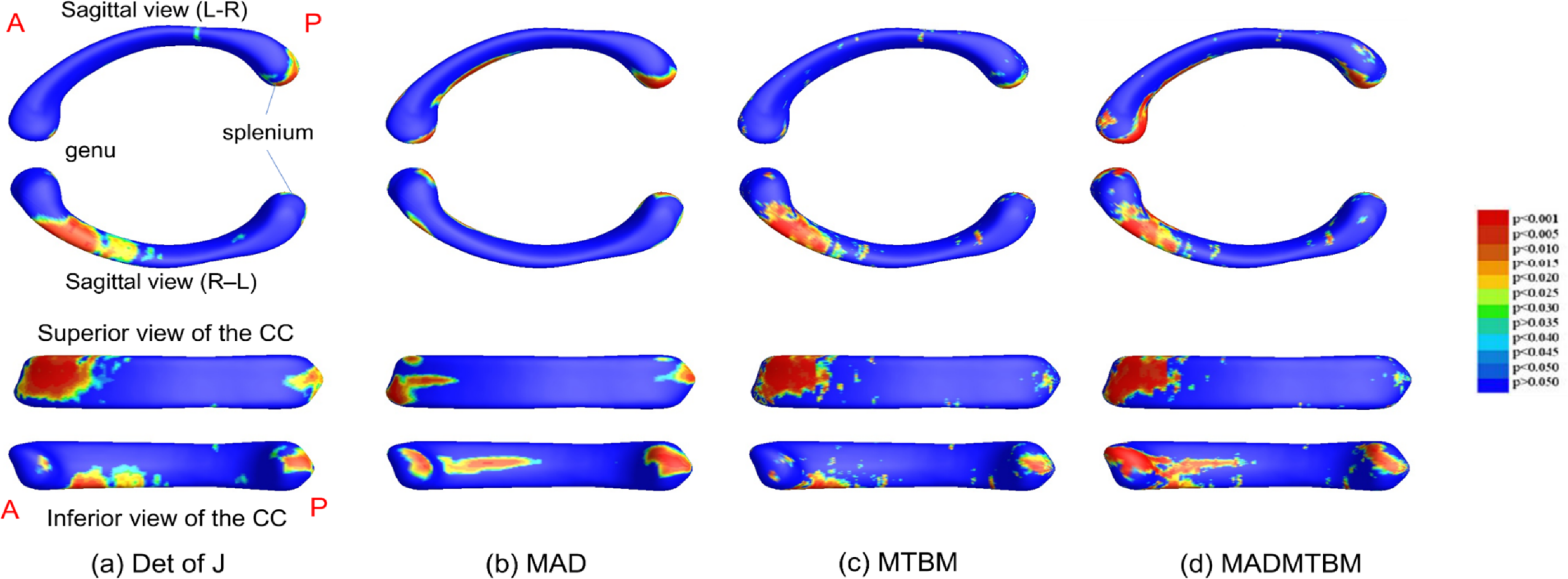
Results showing morphometric differences in the corpus callosum, quantified using (a) the dett of J, (b) MAD, (c) mTBM, and (d) MADMTBM between the 12 m vs 24 m groups.

**FIGURE 3:**
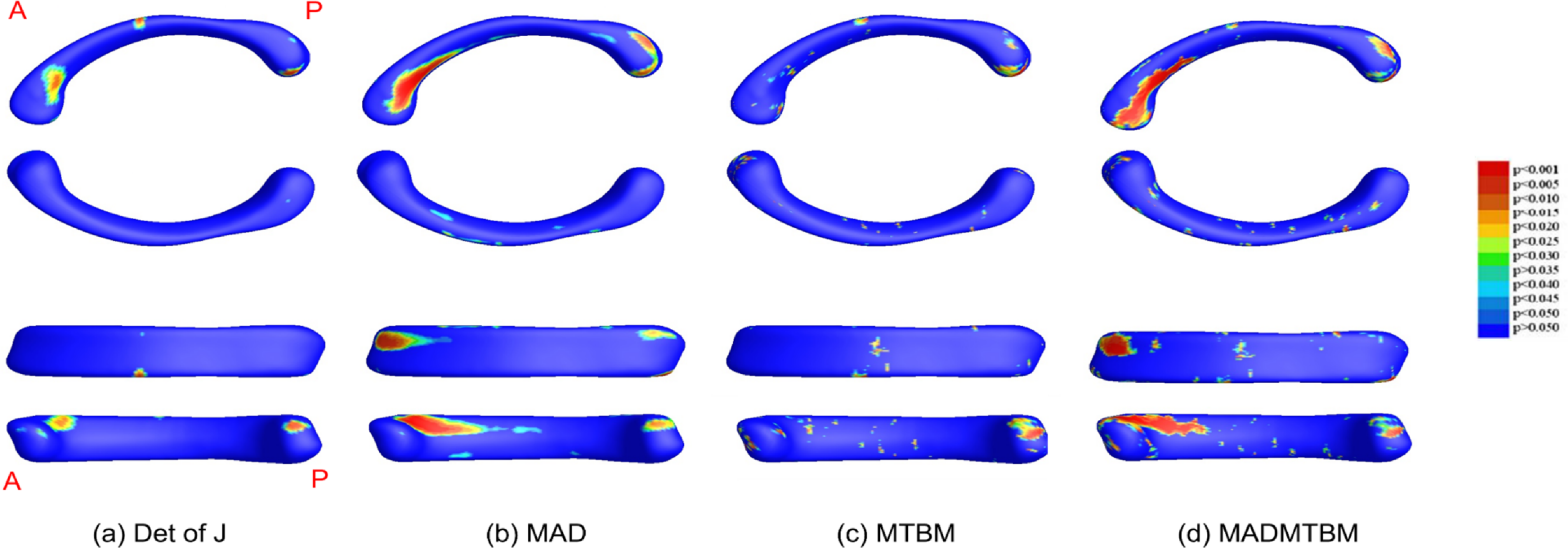
Results showing morphometric differences in the corpus callosum, quantified using (a) the det of J, (b) MAD, (c) mTBM, and (d) MADMTBM between the 24 m vs 36 m groups.

**FIGURE 4:**
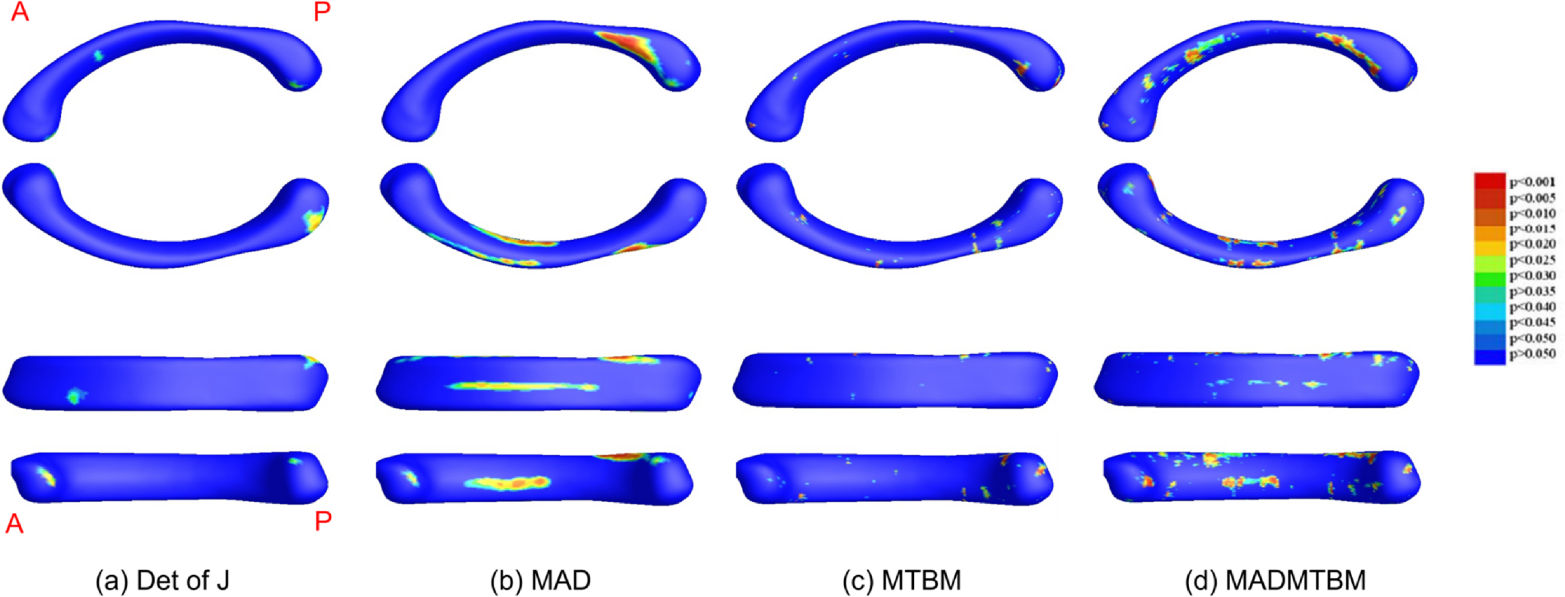
Results showing morphometric differences in the corpus callosum, quantified using (a) the det of J, (b) MAD, (c) mTBM, and (d) MADMTBM between the 36 m vs 48 m groups.

The global map significance of univariate and multivariate comparisons for each age comparison is displayed in Figure 2 for the 12 m and 24 m comparison, Figure 3 for the 24 m and 36 m comparison, and Figure 4 for the 36 m and 48 m comparison. In each figure, a. represents the determinant of the Jacobian matrix (left), b. represents medial axial distance (MAD) results (2nd image), c. represents the mTBM result (3rd image), and d. represents the combined measure of MAD and mTBM (MADMTBM) results, (4th image). These results are for both sexes combined. While mTBM and MADMTBM are more sensitive to localized differences, Det of J and MAD are shown as a reference. Specifically, MAD was used to investigate the directionality of change.

**FIGURE 5:**
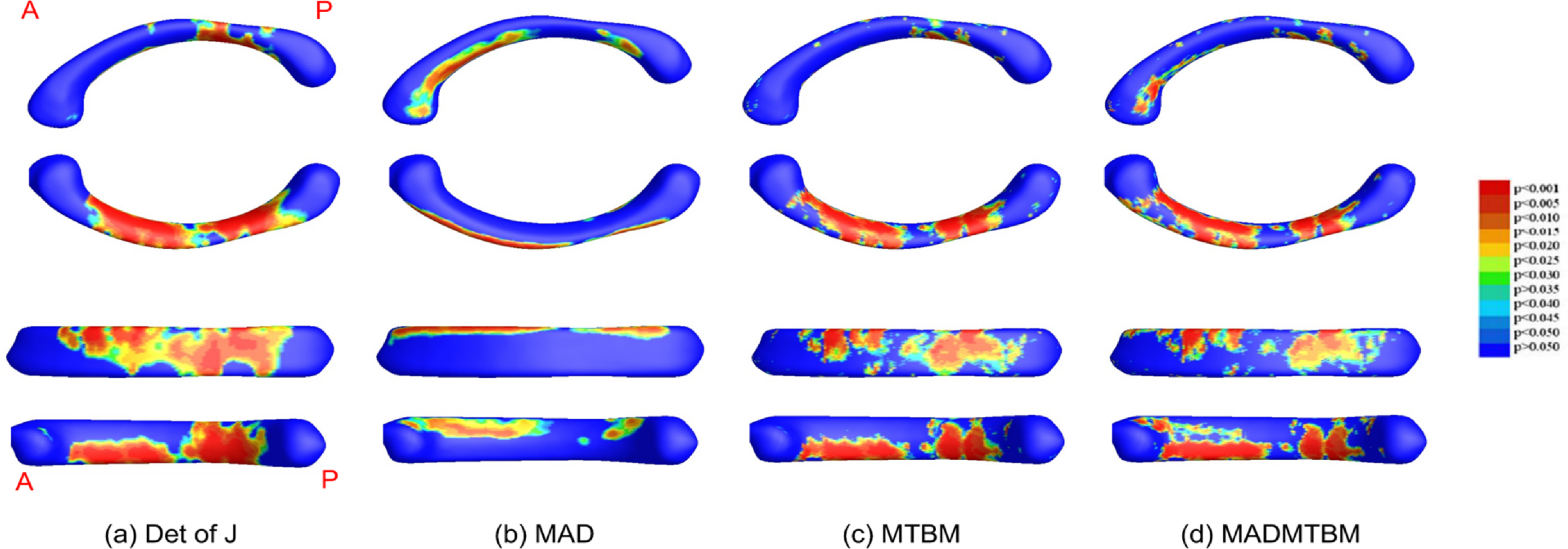
Results showing morphometric differences in the corpus callosum, quantified using a. the det of J, b. MAD, c. mTBM, and d. MADMTBM between the 48 m vs 60 m groups.

**FIGURE 6:**
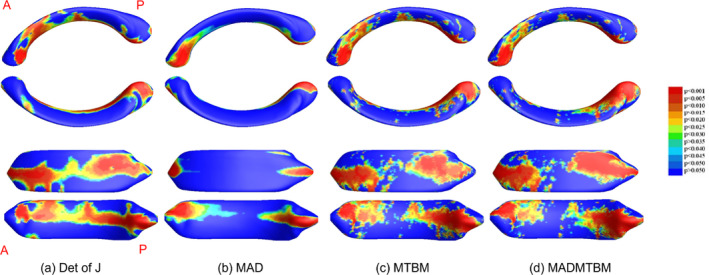
Results showing morphometric differences in the corpus callosum, quantified using (a) the det of J (left top), (b) mTBM (left bottom), (c) MAD (right top), and (d) MADMTBM (right bottom) between the 12 m vs 60 m groups.

The greatest area of significance between groups can be seen in the body of the CC, specifically the anterior midbody in the comparisons between 12 m and 24 m, and the anterior, central, and posterior midbody and isthmus in the comparison between 48 m and 60 m of age (Figures 1 and 5). Additionally, smaller regions of significance were observed in the splenium of the CC (Figure 2) between 12 m and 24 m. The most significant differences in the “middle” age groups (the 24 m and 36 m comparison and the 36 m to 48 m comparison) appear to be more localized to the rostral and anterior mid‐body. Figure 6 shows the area of largest change between the youngest and oldest ages in our cohort. We see that the majority of the body of the CC undergoes significant change.

**FIGURE 7:**
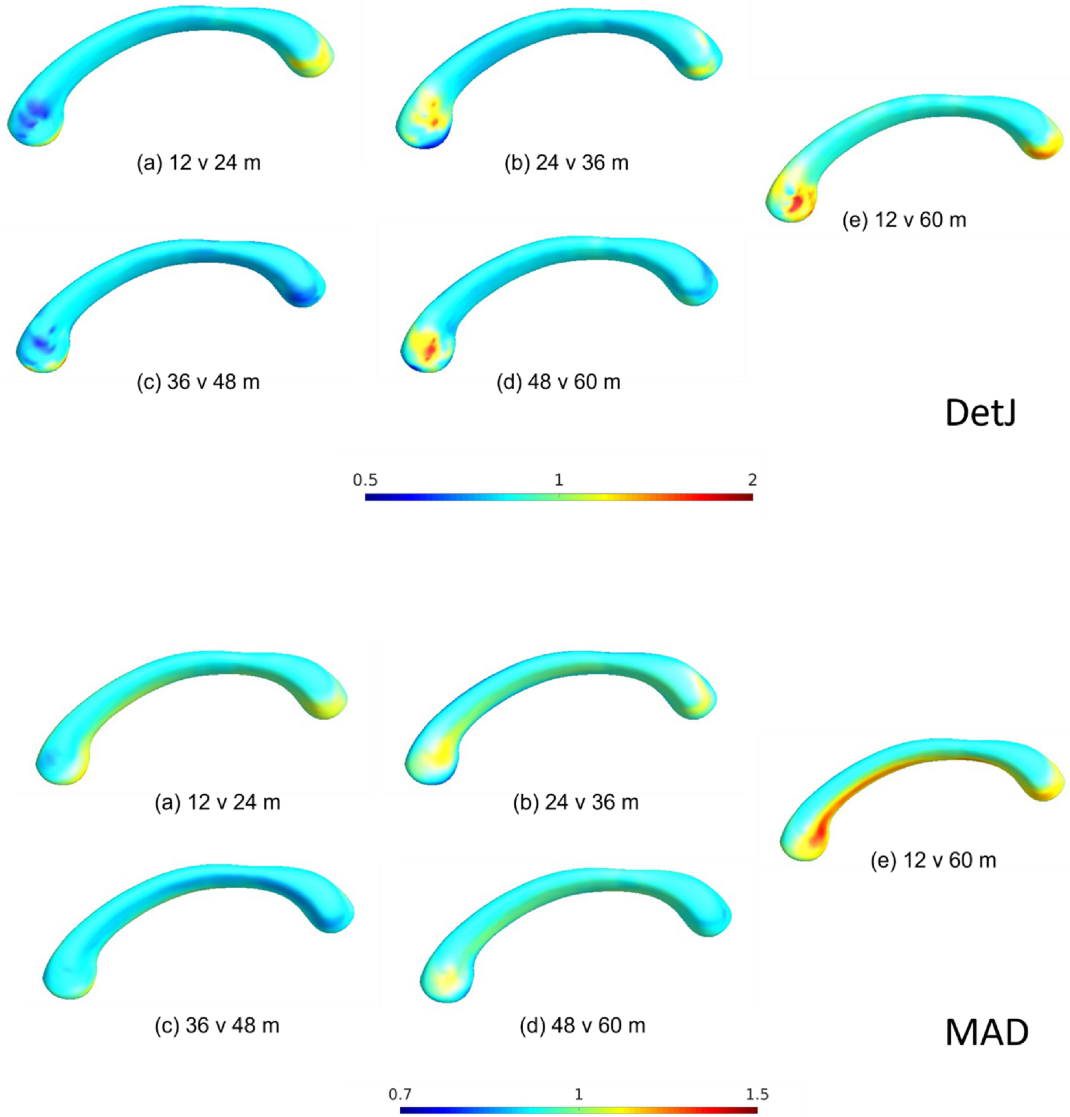
Ratio maps showing the direction of change for Det of J (top) and MAD (bottom). Values of >1 show expansion from the younger to the older age and for Det of J and similarly MAD shows increased distance from the medial axis. Values of <1 indicate a reduction from the younger to the older groups for the Det of J measure and decreased medial axis distance for MAD.

The ratio maps between consecutive ages are shown in Figure 7 to determine the direction of change. In both det of J and MAD, the mean ratio is around 1 between consecutive ages, with expansion in the genu region and to a lesser extent in the splenium region of the CC. We also observed a reduction in the genu at 24 m compared to 12 m and at 48 m compared to 36 m.

**FIGURE 8:**
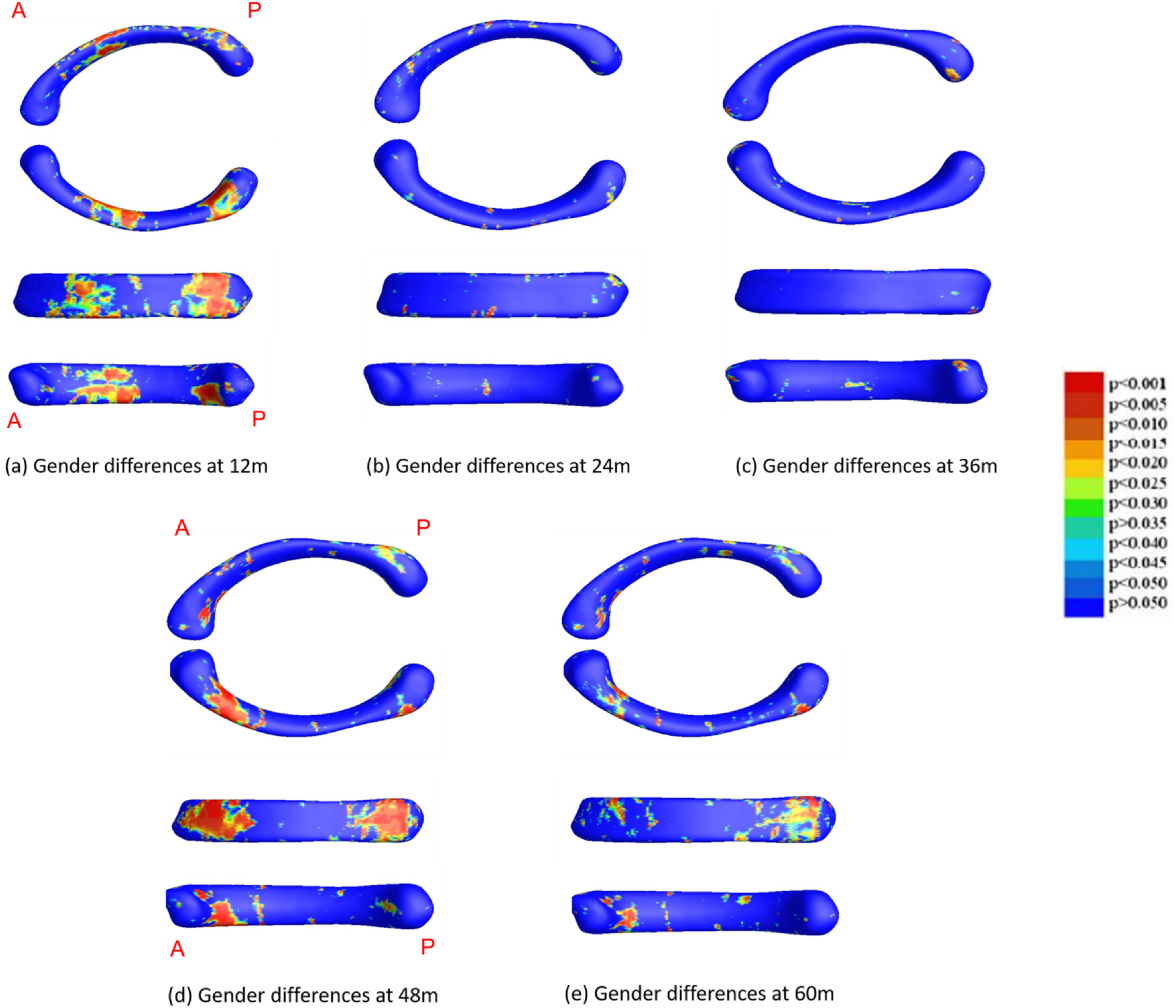
Results for sex differences in corpus callosum development in the 12 m, 24 m, 36 m, 48 m, and 60 groups, as computed using the MADMTBM method due to its superior sensitivity in detecting changes.

Figure 8 shows sex differences in each group as computed using the MADMTBM method due to its superior sensitivity to changes. The most significant difference we see is in the body of the CC between males and females in the 12 m and 48 m cohorts. A cluster of significance is also seen closer to the splenium of the CC between males and females in the 48 m group, with some significance in the 60 m group as well.

## 4 DISCUSSION

In this study, we applied a combined univariate and multivariate measures of the morphological changes of the CC in early development. This is the first time a surface‐based morphometry analysis of the corpus callosum has been done in children between the ages of 12 to 60 months. We note here that all measures (i.e., the det of J, MAD, mTBM, and MADMTBM) are consistent in showing similar regions of significance within the CC. Our main result is by the MADMTBM method, and the other tests are used to confirm the consistency and interpret the results of MADMTBM. While MTBM and MADMTBM are more sensitive to localized differences, we included the det of J and MAD as references, and to investigate the directionality of change. MADMTBM showed the most exhaustive changes between the groups suggesting higher detection power and largest sensitivity to local surface changes of the CC, thus allowing a better characterization of the morphological changes. Our results indicate that while most of the CC changes significantly between 12 and 60 months of age, the most prominent differences detected are in the anterior and posterior parts of the body of the CC, especially between the 48 m and 60 m groups. This is consistent with Giedd et al., who noted that while the midsagittal area of the corpus callosum increased in a cohort of children aged 5–18 years, significant increases were seen in the mid and posterior regions (Giedd, Blumenthal, Jeffries, Castellanos, et al., 1999; Giedd, Blumenthal, Jeffries, Rajapakse, et al., 1999). Ratio maps for the det of J and MAD measures show a large part of these changes are gradual expansion.

As a child grows, changes are observed throughout the CC. These differences may be attributed to a variety of factors, including normal brain growth, gradual increases in myelination occurring directionally from the caudal to the rostral regions, motor and sensory development, environmental factors, and synaptic pruning (Krupa & Bekiesinska‐Figatowska, 2013). When considering developmental changes in the CC, it is important to acknowledge the influences of connectivity patterns and specific localization of fiber projections throughout the CC. Studies in animal models have helped us delineate the topographic organization of corpus callosum fiber projections and further confirmed that callosal projection cells progressively extend across the cerebral cortex in both the later‐to‐medial and rostral‐to‐caudal directions (Ozaki & Wahlsten, 1998; Olivares et al., 2001; Caminiti et al., 2021; Innocenti et al., 2022). Furthermore, DTI tractography studies of WM fiber projections from the CC have shown that the fibers from the genu project to the prefrontal lobe, and fibers from the CC body project to the premotor and supplementary motor areas, the primary motor cortex, and primary sensory regions (Fabri & Polonara, 2023). The remaining part of the CC, including the splenium, projects fibers to the parietal, occipital, and temporal lobes (Hofer & Frahm, 2006). We consider these findings in interpreting our results.

Between 12 versus 24 months of age, we see significant differences in the anterior midbody of the CC, in regions corresponding to premotor fiber projections, which may indicate development of movement planning behavior in children. This is congruent with the Bayley Scales of Infant Development and the Infant Development Inventory, both of which state that the period between 12 months and 24 months is accompanied by increasingly coordinated movements of both fine and gross motor skills. Significance in the splenium regions is consistent with a post‐mortem study that showed the splenium was myelinated in 90% of autopsied cases in the study by 21 months of age (Brody et al., 1987). The white matter fibers from the splenium connect to regions of the temporal and occipital lobes as shown in previous DTI studies (Hofer & Frahm, 2006). The significant difference observed in this region might reflect the early changes associated with the visual system development known to mature early in childhood (Gilles & Nelson, 2012).

The lateral sides of the body of the CC change significantly between 48 versus 60 m; fibers from these regions of significant difference likely project to the premotor, sensory, motor, parietal, and temporal regions of the cortex (Schmied et al., 2020), indicating intense development of these regions. This result is also consistent with Giedd et al., describing the increase in total CC midsagittal area. Westerhausen et al. showed that posterior CC regions (especially the splenium) increase linearly compared to all middle and anterior segments, in children between the ages of 4 and 11 (Westerhausen et al., 2016). While we did not see significant differences in the splenium between the 48 and 60 m comparison, it is possible that changes may appear later than 60 months. Additionally, not all changes may be captured as significant due to high variability in childhood brains.

In contrast, between 24 versus 36 months and 36 m 48 m of age, differences were more subtle and limited to a smaller region in the body of the CC, a region likely projecting fibers to the pre‐motor and supplementary cortices. This indicates the ongoing development of early movement planning behavior in children. From various literature on development (Giedd & Rapoport, 2010; Li et al., 2014, 2014; Robinson & Proctor, 2009; Zhang et al., 2015) as well as studies on CC development (Deoni et al., 2012; Fujii, Kuriyama, Konishi, Saito, & Sudo, 1994; J N Giedd, Blumenthal, Jeffries, Castellanos, et al., 1999; Gilliam et al., 2011; Schmied et al., 2020; Tanaka‐Arakawa et al., 2015; Vannucci, Barron, & Vannucci, 2017), we would expect to see larger clusters in these age ranges. However, as surface‐based morphometry has not been used previously, the differences seen in mid‐sagittal area, diffusion, or volume may not be necessarily reflected in morphometric analysis. Due to the rapid development of the brain in early childhood, variability in the data can be a limiting factor and may result in subtle differences not being detected. Additionally, since the body of the CC is heavily myelinated by 12 months of age (Gilles & Nelson, 2012) significance may only be achieved when there is a large amount of change. Another limiting factor in this study is the binning method used. We used a 6 month binning interval to accommodate the different dynamics of the developing brain under the age of 24 months (Gilmore et al., 2018), and a 12 month binning interval for the older age groups. However, this binning interval may be crude and future directions may include developing methods that can capture differences on the order of days or weeks.

Previous studies of the CC used tensor maps, indicating radial tissue expansion rates, to determine a subject's growth between 3 to 6 years (Thompson et al., 2000). The authors showed peak growth in the anterior region of the CC, which projects to the frontal circuits and is involved in organization and planning of actions. Additionally, myelination studies done in all WM have shown exponential increase in the myelin water fraction (MWF) in the first 500 days in all parts of the CC, after which myelination increased more gradually (Dean et al., 2014). The significant differences we see in the body of the CC in our comparisons may reflect this radial tissue expansion and the exponential increase captured by the MWF.

We also investigated sex differences within each group. The largest differences we see are at 12 m, 48 m, and 60 m in the anterior part of the body and the posterior part of the CC, including isthmus region of the CC. However, little difference was observed in the other age groups. Sex differences are challenging to pinpoint, perhaps due to the dynamic changing nature of the infant brain, the size of the dataset needed, and the types of analysis performed. Giedd et al. observed no significant effects of sex for the midsagittal area measures used in their study, involving children above age 4 (J N Giedd et al., 1996). A study involving infants between the ages of 6 and 24 months showed sex differences in the CC area and thickness adjusted for brain size, where males had a larger growth rate compared to females (Schmied et al., 2020). Another study has shown that females had a higher splenial length/height ratio as well as a hemispheric length/height ratio compared to males between the ages of 0–24 month, indicating a longer female brain in infancy (Vannucci et al., 2017). These differences were shown to be disappearing with age (Vannucci et al., 2017). Due to the analysis method and the ages involved, we cannot directly compare our results with previous literature. However, the innovative analysis of MADMTBM allows us to pinpoint subtle differences that would have been difficult to identify previously without a large dataset.

## 5 CONCLUSION

The CC is an important structure to characterize due to the role it plays in the brain and in brain development. In this study, we showed that using combined measures of multivariate mTBM and MAD, we could identify localized differences in the 3D structure of the CC in brain development between the ages of 12 and 60 months. These differences were more pronounced in the body of the CC. Additionally, sex‐based differences were also investigated, and we were able to observe localized differences within each group using MADMTBM. Surface‐based morphometry utilizing the 3D structure information adds to the body of literature investigating the growth of the CC and sex differences in early childhood. Future studies will include creation of developmental trajectories as well as include more ages to get a full range of subjects.

## CONFLICT OF INTEREST STATEMENT

None of the authors have a conflict of interest to disclose.

## Data Availability

The data that support the findings of this study are available on request from the corresponding author. The data are not publicly available due to privacy or ethical restrictions.
